# Supermicrosurgical lymphatic venous anastomosis for intractable lymphocele after great saphenous vein harvesting graft

**DOI:** 10.1016/j.jvscit.2021.11.003

**Published:** 2021-11-22

**Authors:** Hirofumi Imai, Shuhei Yoshida, Toshiro Mese, Solji Roh, Isao Koshima

**Affiliations:** International Center for Lymphedema, Hiroshima University Hospital, Hiroshima, Japan

**Keywords:** Great saphenous vein harvesting, Lymphatic venous anastomosis, Lymphorrhea

## Abstract

Lymphoceles result from either trauma to the lymphatic vessels or after vein graft harvest, which occurs in 10% to 16% of patients. When a lymphocele persists despite conservative treatment, patients can experience undue distress. We have reported the case of successful treatment using lymphatic venous anastomosis (LVA) of an intractable lymphocele that had been refractory to conservative treatment, including stretch bandaging, drainage, and local injection for 2 years after great saphenous vein harvest. The lymphocele resolved shortly after the LVA without any adverse effects. LVA can be a useful and minimally invasive alternative treatment of lymphocele after harvesting the great saphenous vein.

A lymphocele can occur after trauma to the lymphatic vessels, and 10% to 16% of patients will develop lymphoceles or lymphorrhea after vein graft harvest.^1^ Large lymphatic vessels lie adjacent to the great saphenous vein and are likely to be injured during vein grafting.^2^ When a lymphocele persists despite conservative treatment, including stretch bandaging, drainage, and sclerotherapy, patients can experience undue distress from the uncontrollable lymphocele.

Lymphatic venous anastomosis (LVA) is a surgical treatment that improves lymphatic drainage by anastomosing the lymphatic vessels to a cutaneous vein under surgical microscopy. LVA is one of the alternative methods for the treatment of lymphoceles and lymphorrhea.^3^^,^^4^

In the present report, we have described a rare case of lymphocele that had developed as a postoperative complication of great saphenous vein graft harvest. Treatment was successful using LVA. The institutional review board of Hiroshima University Hospital approved the present study. The patient provided written informed consent for the report of his case and imaging studies.

## Case report

A 70-year-old male patient had undergone renal transplantation after renal failure, with his great saphenous vein harvested for the transplant. He had a history of glomerulonephritis since childhood and had undergone renal transplantation at 68 years of age. During his renal transplantation, the great saphenous vein was harvested from the right thigh to the groin. The patient had been receiving immunosuppressive drugs since then. After the great saphenous vein was harvested, the patient had developed a hydrocele under the dermis of the medial side of the right thigh. Approximately 30 mL of fluid was aspirated from the hydrocele once every 2 to 3 days for 2 years. After treatment using local injection, including glucose and minocycline, at the site of the hydrocele had failed, the patient visited our hospital for the treatment of an intractable hydrocele.

**Fig 1:**
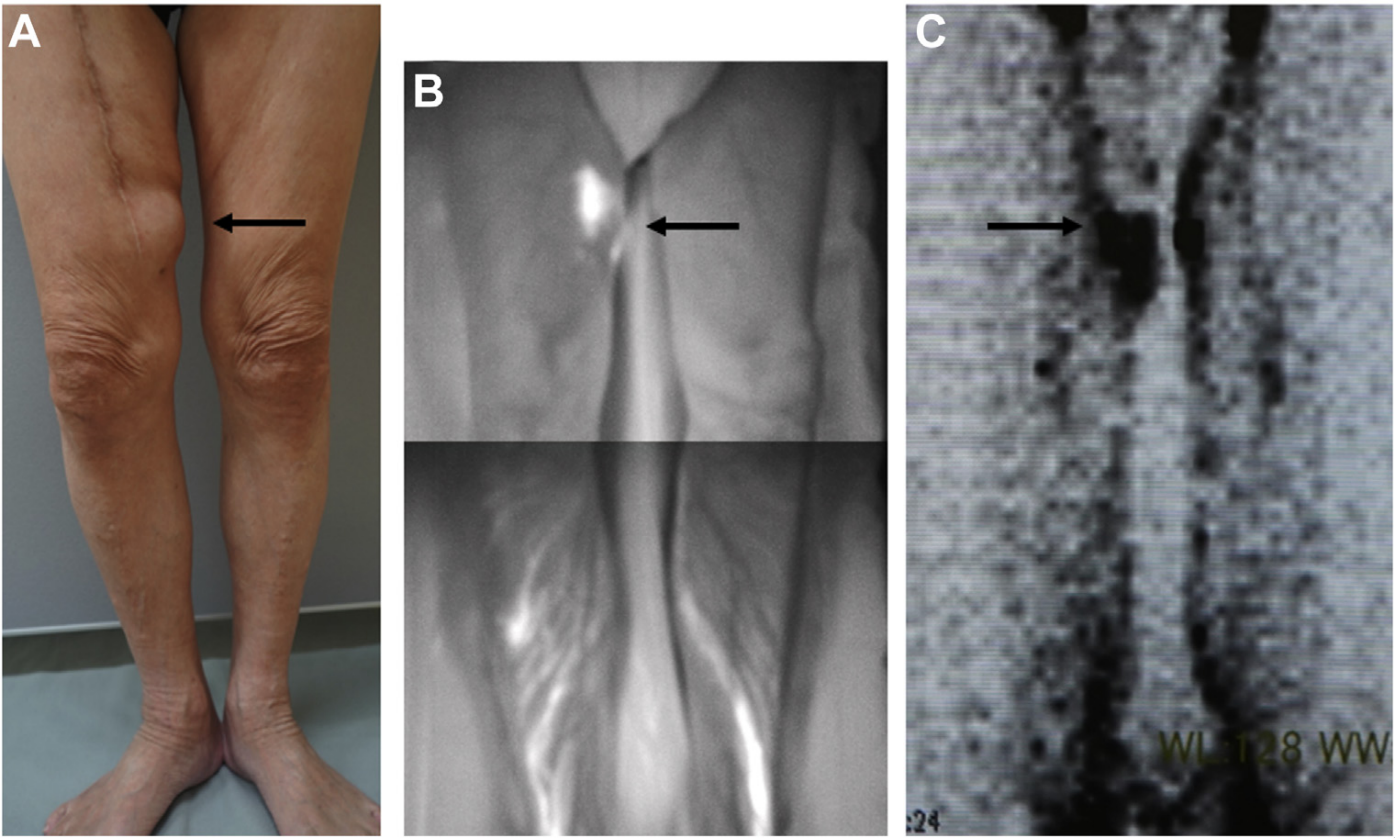
**A,** Photograph of the patient's lower extremities showing the lymphocele (*arrow*) in the medial side of the thigh along the scar where the great saphenous vein had been harvested. Indocyanine green lymphography **(B)** and lymphoscintigraphy **(C)** revealed pooling (*arrow*) at the site of the lymphocele.

The patient had a protuberance over the medial side of the thigh along the scar at the site where the great saphenous vein had been harvested (Fig 1, *A*). An ultrasound study revealed a 3.0 × 3.0-cm-size hydrocele containing 35 mL of clear yellow serum, which was confirmed by fine needle aspiration. The indocyanine green lymphography and lymphoscintigraphy findings demonstrated pooling on the medial side of the right thigh (Fig 1, *B* *and* C) and a lymphocele was diagnosed. After puncture of the lymphocele, we attempted treatment with a high pressure bandage; however, the lymphocele had recurred after 3 days. Thus, to treat the intractable lymphocele, we performed LVA to drain the lymph into the vein.

**Fig 2:**
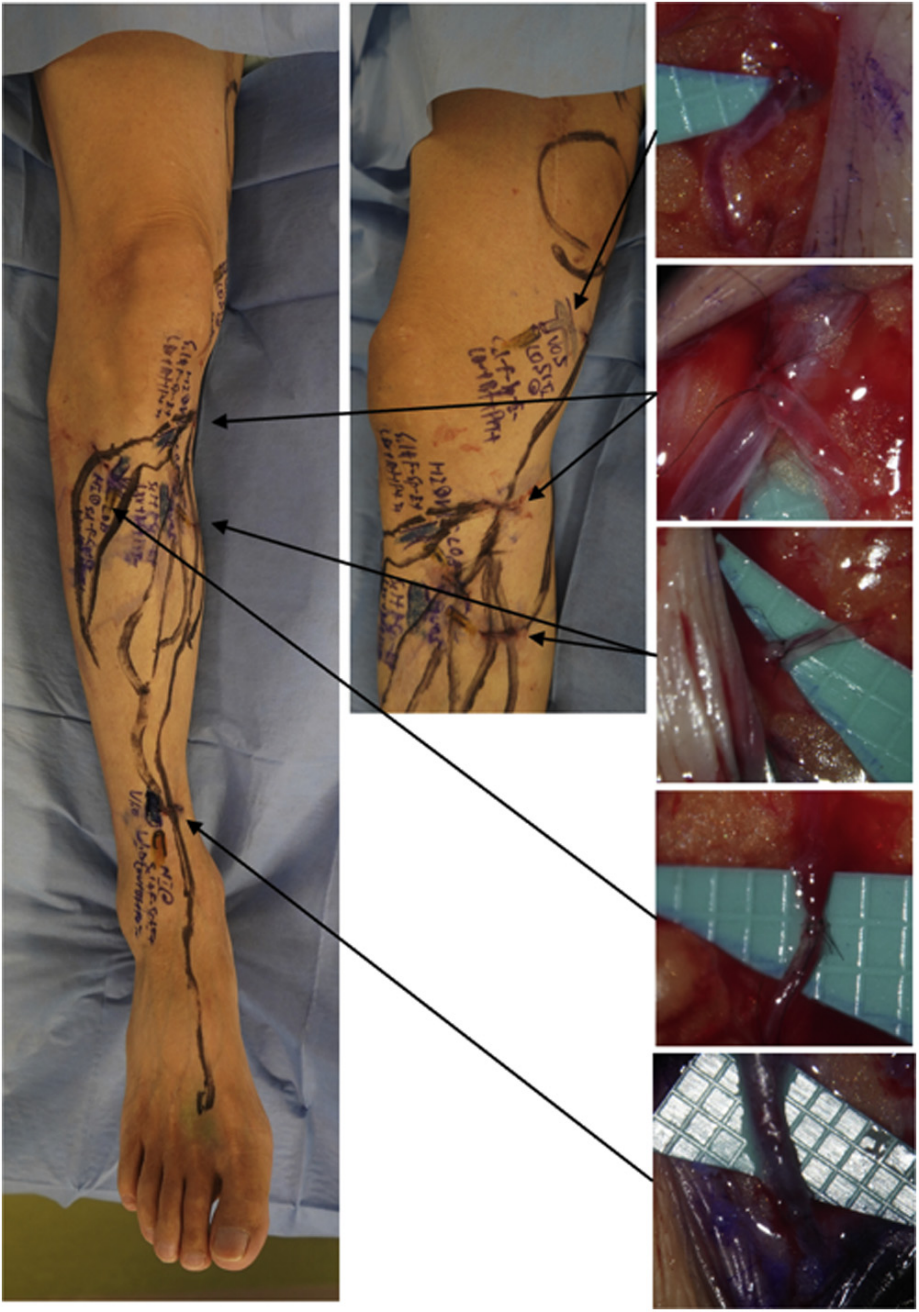
Operative findings for lymphatic vein anastomosis (LVA). One LVA was performed on the medial side of the right thigh, and four were performed in the right lower leg.

**Fig 3:**
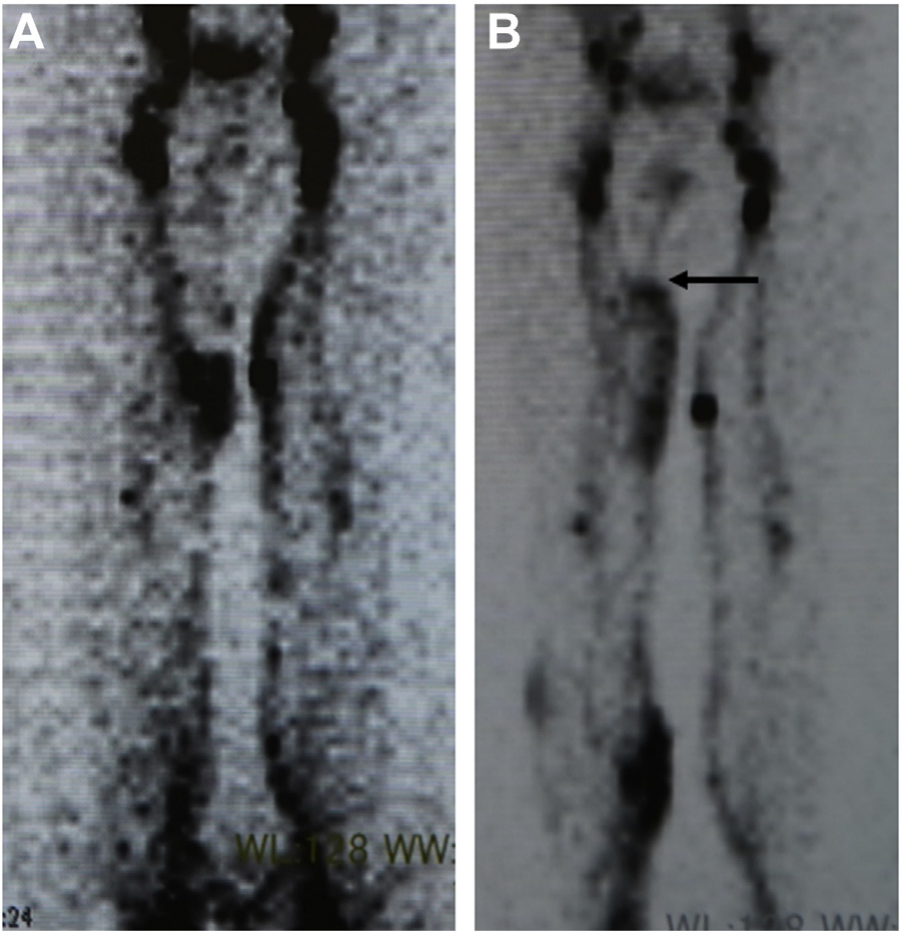
Comparison of lymphoscintigraphy before and after surgery. **A,** Lymphoscintigraphy before surgery. **B,** After surgery, lymphoscintigraphy demonstrated upstream lymph flow over the site of the lymphocele, with several pathways found over the proximal side of the right medial thigh (*arrow*).

One LVA was performed on the medial side of the right thigh and four LVAs were performed in the right lower leg (Fig 2). Using 12-0 needles, 50 μm in diameter, one end-to-side and four end-to-end LVAs were performed. The lymphocele gradually became smaller and had disappeared 4 weeks postoperatively without any punctures required. The patient did not wear the bandage after the operation. Eight months after surgery, lymphoscintigraphy had demonstrated lymph fluid upstream over the site of the lymphocele, and several pathways were found on the medial side of the right thigh, a greater number than that those observed preoperatively (Fig 3). No recurrence of the lymphocele had been reported at 15 months postoperatively.

## Discussion

We have described a single case demonstrating the effectiveness of LVA in treating intractable lymphoceles that have been refractory to conservative treatment. Our patient had undergone stretch bandaging, drainage, and local injections for 2 years after the great saphenous vein had been harvested. Lymphocele and lymphorrhea will occur in 10% to 16% of patients after a great saphenous vein graft has been harvested.^1^ In addition, our patient had been receiving immunosuppressive drugs, which correlated with the occurrence of peripheral lymphedema.^5^^,^^6^ Intractable lymphocele at the site where a vein was harvested is relatively uncommon and has generally been underrated by physicians, increasing the risk of morbidity for patients. However, when it occurs, it can be difficult to manage. Furthermore, disruption of the lymphatic vessels during vein harvesting has been associated with postoperative lymphedema after harvesting of the great saphenous vein.^7^

Radical treatment of intractable lymphocele has not been established. A variety of conservative treatments, including stretch bandages, drainage, and local injections, has been used.^8^ However, for some patients, conservative treatment will fail. Lymphatic embolization and macroscopic ligation of the ruptured lymphatic vessels in the subcutaneous fat have been performed. However, disruption of lymph drainage can result in lymphedema.^9^^,^^10^ LVA, which allows for the flow of lymph into the venous circulation, is considered the most appropriate therapeutic approach for lymphedema because it addresses the pathophysiology of the condition. Yamamoto et al^4^ and Yoshida et al^11^ performed LVA to treat lymphorrhea accompanied by genital lymphedema and resection of soft tissue sarcomas, respectively, and reported that the lymphorrhea had resolved soon after the LVA. LVA has been used as an alternative treatment of lymphocele and lymphorrhea. However, to the best of our knowledge, no study has reported using LVA to treat a lymphocele at the graft harvest site. In the present patient, multisite LVA was performed, although microsurgical ligation of the disrupted lymphatics was considered because of the ligation-induced lymphedema, which had possibly resulted from the immunosuppressive drugs.^5^^,^^6^^,^^8^^,^^9^ It could also have been completed as a single LVA procedure with good results for the lymphocele; however, multisite LVA can be more effective and therapeutic for lymphedema.^12^ To divert the flow of the lymphocele-causing lymph into the venous circulation, we performed multisite LVA by targeting the lymph channel linked to the lymphocele as shown by indocyanine green lymphography findings.

Lymphoscintigraphy can help to diagnose, as well as treat, lymphocele. In case of a lymphocele, lymphoscintigraphy will enable successful visualization of the leakage.^13^ Lymphoscintigraphy demonstrated pooling on the medial side of the right thigh along the harvest site of the great saphenous vein. However, upstream lymph flow toward the site of the lymphocele and several pathways were found over the medial side of the right thigh, greater than the number observed preoperatively. These findings suggest that the flow of lymph into the venous circulation by the LVA had decreased the subcutaneous lymphocele, with lymph flow reactivation at the proximal medial side of the thigh resulting from resolution of the lymphocele.

## Conclusions

We performed LVA after failure of conservative treatment of a lymphocele. The surgery was successful with no adverse effects within the patient's postoperative course. LVA has the potential to be the first-line treatment of intractable lymphoceles resistant to conservative treatment owing to its minimally invasive approach. However, the present report was limited to a single case. Additional studies with a larger number of patients are required to prove the efficacy of LVA for the treatment of intractable lymphoceles after harvest of the great saphenous vein.
